# Effect of Labeling with Iron Oxide Particles or Nanodiamonds on the Functionality of Adipose-Derived Mesenchymal Stem Cells

**DOI:** 10.1371/journal.pone.0052997

**Published:** 2013-01-03

**Authors:** Sinead P. Blaber, Cameron J. Hill, Rebecca A. Webster, Jana M. Say, Louise J. Brown, Shih-Chang Wang, Graham Vesey, Benjamin Ross Herbert

**Affiliations:** 1 Department of Chemistry and Biomolecular Sciences, Macquarie University, North Ryde, New South Wales, Australia; 2 Regeneus Ltd, Gordon, New South Wales, Australia; 3 Department of Physics and Astronomy, Macquarie University, North Ryde, New South Wales, Australia; 4 Department of Radiology, Westmead Hospital, University of Sydney, Westmead, New South Wales, Australia; University of Medicine and Dentistry of New Jersey, United States of America

## Abstract

Stem cells are increasingly the focus of translational research as well as having emerging roles in human cellular therapy. To support these uses there is a need for improved methods for *in vivo* cell localization and tracking. In this study, we examined the effects of cell labeling on the *in vitro* functionality of human adipose-derived mesenchymal stem cells. Our results provide a basis for future *in vivo* studies investigating implanted cell fate and longevity. In particular, we investigated the effects of two different particles: micron-sized (∼0.9 µm) fluorescently labeled (Dragon Green) superparamagnetic iron oxide particles (M-SPIO particles); and, carboxylated nanodiamonds of ∼0.25 µm in size. The effects of labeling on the functionality of adipose-derived MSCs were assessed by *in vitro* morphology, osteogenic and adipogenic differentiation potential, CD marker expression, cytokine secretion profiling and quantitative proteomics of the intra-cellular proteome. The differentiation and CD marker assays for stem-like functionality were not altered upon label incorporation and no secreted or intra-cellular protein changes indicative of stress or toxicity were detected. These *in vitro* results indicate that the M-SPIO particles and nanodiamonds investigated in this study are biocompatible with MSCs and therefore would be suitable labels for cell localization and tracking *in vivo*.

## Introduction

Regenerative medicine is a rapidly emerging field with a major focus on stem cell transplantation in experimental animal models. Historically, stem cell research has focused on *ex vivo* differentiation of mesenchymal stem cells (MSCs) into cells of the target tissue type or *in vivo* engraftment and differentiation as the key drivers of therapeutic tissue repair. These modes of action localized to the site of tissue damage and cell tracking is not a major consideration. However, the therapeutic properties of MSCs have been studied extensively *in vitro* and *in vivo* and it is now clear that MSCs are able to induce tissue repair without differentiation. They are mobile after implantation and have a number of modes of action including immuno-modulation, angiogenic, anti-apoptotic and anti-scarring properties through paracrine signalling [1]–[8]. MSCs delivered at remote sites, home to the site of injury where they engraft, control the microenvironment and stimulate the endogenous cells to repair and regenerate the damaged tissue [9]. However, to gain some insight into cell migration, tissue localization, the level of engraftment, or the longevity of these cells following implantation, the cells require labeling and subsequent tracking.

The ability to track cells *in vivo* in a non-invasive manner with repeated imaging is advantageous for animal model studies given that the majority of the traditional techniques for determining the fate of labeled cells involve postmortem histological analysis. Repeat imaging on live animals enables time course data to be collected with fewer animals. In human clinical studies the use of biocompatible labels that enable MRI imaging is likely to assist in the refinement of cell therapy by enabling cell fate and localization data to be correlated with therapeutic outcome measures. There are a number of ways to achieve labeling of cells, of which fluorescent and/or magnetic labels are the most extensively used. The imaging of fluorescently labeled cells most often requires tissue sampling for detection, however new imaging machines have recently been developed for the live tracking of fluorescently labeled cells in small animals, such as the *in vivo* FX PRO (Carestream, USA). Magnetic particles for the labeling of cells are also particularly attractive because they can be imaged non-invasively in real-time using magnetic resonance imaging (MRI). Superparamagnetic iron oxide particles (SPIO) are usually used for cell labeling due to their biocompatibility with cells and their strong effects on spin-spin relaxation time (T_2_) and on the corresponding transverse relaxation time constant (T_2_^*^) during MRI imaging [10]–[13].

In recent years, nanodiamonds have also emerged as important particles for a variety of bioapplications including the development of therapeutic agents for diagnostic probes, gene therapy, targeted delivery vehicles, anti-viral and anti-bacterial treatments, tissue scaffolds, protein purification and labeling of cells for tracking [14]. Nanodiamonds are attractive particles for these bioapplications because they possess important properties such as biocompatibility and a surface structure that can be easily modified to facilitate bioconjugation. Furthermore, nanodiamonds have highly stable photoluminescence properties and can be produced in a range of sizes; and inexpensively, on a large scale [15].

Regardless of which type of particle is used for the labeling and subsequent tracking of cells, it is important to determine what effect, if any, the labeling procedure has on the function of the cells. It is imperative to ensure that the results obtained from the *in vivo* tracking of labeled cells are not due to any label-induced alteration in cell function. As MSCs are increasingly the focus of *in vitro* and *in vivo* research as well as having emerging roles in human cellular therapy, there is also the need for improved methods for cell localization and tracking. In this study, we examined the effects of cell labeling on the functionality of human adipose-derived MSCs *in vitro*. Our results provide a basis for future in *vivo* studies investigating MSC cell-fate and longevity. In particular, we investigated the effects of two different particles: micron-sized (∼0.9 µm) fluorescently labeled (Dragon Green) superparamagnetic iron oxide particles (M-SPIO particles); and, carboxylated nanodiamonds of ∼0.25 µm in size. The effects of labeling on the functionality of adipose-derived MSCs were assessed by *in vitro* morphology, differentiation potential, CD marker expression, cytokine secretion profiling and quantitative proteomics of the intra-cellular proteome.

## Materials and Methods

### Isolation and expansion of MSCs from a human lipoaspirate

This research was approved by the Macquarie University human research ethics committee (Ref #: 5201100385). Written consent was obtained from the patients who participated in this study. A human abdominal lipoaspirate was obtained from a patient undergoing routine liposuction procedures for cosmetic reasons and processed as previously described [16]. Briefly, 200 g of the lipoaspirate was digested with 0.5 mg/mL collagenase (Lomb Scientific, USA) in saline supplemented with 0.05 mg/mL of vancomycin (Hospira Australia Pty Ltd, Australia) in a 37°C water bath for 30 mins with periodic mixing. The digested sample was passed through an 800 µm mesh and centrifuged at 1500×*g* for 5 mins to obtain the pelleted cells (SVF) and floating adipocytes. The floating adipocytes were discarded. The SVF fraction was washed with saline, centrifuged at 1500×*g* for 5 mins and seeded into T175cm^2^ flasks containing Standard Media that consisted of Dulbecco's Modified Eagle Medium (DMEM; Life Technologies, USA) supplemented with 10% foetal bovine serum (FBS; Bovogen, Australia) and 1% Penicillin-Streptomycin solution (Life Technologies, USA). Media changes were performed every 3 days. The initial media change resulted in removal of non-adherent cells. Once the adherent cells (MSCs) reached 80% confluency, cells were washed with DMEM and passaged using TrypLE express (Life Technologies, USA). MSCs were used at passage 2 for the experiments described in this manuscript unless otherwise stated.

### Labeling of adherent MSCs with M-SPIO particles and nanodiamonds

The M-SPIO particles used in this study were ∼0.9 µm diameter superparamagnetic microspheres with a polystyrene coating incorporating dragon green fluorophore (ex480, em520) purchased from Bangs Laboratories, USA. As reported by the manufacturer, the M-SPIO particles may have some level of iron exposure on their surface.

The nanodiamond particles were produced from 0.5 µm ProSciTech diamond powder using extensive acid washing and ultrasonication to remove surface graphite and deaggregate the particles. Briefly, the diamond powder was refluxed in a 9∶1 ratio of concentrated sulphuric to nitric acids for 1 day at 70°C followed by ultrasonication in deionized water with a horn-type sonotrode (Branson Sonifier, USA) for 1 hour. The diamond particles were then acid refluxed for an additional 3 days, rinsed and ultrasonicated for 1 hour in 0.1 M sodium hydroxide. The diamond particles were subsequently rinsed, ultrasonicated in 0.1 M hydrochloric acid, rinsed and resuspended at a concentration of 0.45 mg/mL in deionized water. The size of the resulting carboxylated diamond particles was then determined using Dynamic Light scattering (Malvern Instruments Zetasizer NanoZS) to be 270±30 nm.

The cells were grown to 80% confluency to which M-SPIO particles or nanodiamonds were added at a volume of 0.104 µL/cm^2^ and 1.045 µL/cm^2^ respectively. This equates to 3.33 µg of M-SPIO particles and 1.50 µg of nanodiamonds per mL of cell culture media. The M-SPIO particles and nanodiamonds were incubated with the MSCs for 3 days to allow incorporation into the cells. After 3 days, the MSCs were washed with DMEM, stripped from the surface using TrypLE express and re-seeded into fresh 6-well plates or T175cm^2^ flasks to ensure the particles had incorporated into the cells. The following experiments were then performed on the labeled cells to assess the impact of the label on the functioning of the MSCs.

### Differentiation of adherent MSCs

Differentiation into osteogenic and adipogenic lineages were performed on human MSCs, MSCs labeled with M-SPIO particles and MSCs labeled with nanodiamonds, as described above. MSCs and labeled MSCs were seeded at a density of 1×10^4^ and 5×10^3^ cells per cm^2^ in 6-well plates for adipogenic and osteogenic differentiation respectively. Defined adipogenic and osteogenic differentiation media formulations were used as previously described [17]. The cells received media changes every 3 days. Upon completion of differentiation, cells were washed twice with PBS and incubated for 30 mins with 4% paraformaldehyde. For adipogenic differentiation, the cells were subsequently washed with MilliQ water, incubated with 60% isopropanol, stained with 0.2% Oil Red O solution for 5 mins at room temperature and washed with tap water. For osteogenic differentiation, the cells were stained with 2% Alizarin red solution for 2 mins at room temperature and washed 3 times with MilliQ water. Control and differentiated cells were imaged using a Carl Zeiss Primo Vert inverted microscope.

### CD marker characterization of adherent cells

Control and labeled MSCs were liberated from the wells using TrypLE express, diluted in Standard Media and centrifuged at 2000×*g* for 5 mins. The cells were washed, resuspended in PBS with 2% FBS and stained with the following antibodies, which were all sourced from Becton Dickinson: CD34-FITC (#555821), CD45-FITC (#555482), CD73-PE (#550257), CD90-FITC (#555595), and CD105-PE (#560839) and incubated on ice for 45 mins. Cells were washed with ice cold PBS, centrifuged at 300×*g* for 5 mins and resuspended in 1× FACS Lysing Solution (Becton Dickinson, USA). The cells stained with FITC conjugated antibodies were resuspended in propidium iodide (10 µg/mL) and isoflow. Stained and unstained control cells were analysed using a FacsScan flow cytometer (Becton Dickinson, USA).

### Preparation of MSCs and labeled MSCs for Bio-Plex Analysis of Human Cytokines and Growth Factors in conditioned medium

To assess the effect of labeling on the secretion profiles of MSCs, two separate seeding experiments were performed. The first involved control MSCs and MSCs labeled with M-SPIO particles, whilst the second involved control MSCs and MSCs labeled with nanodiamonds. In each experiment, 2×T175cm^2^ passage 2 MSCs were split evenly into 6 fresh T175cm^2^ flasks. Of these flasks, 3 served as control MSCs cells and 3 flasks were labeled with either M-SPIO particles of nanodiamonds as described earlier. The cell numbers were normalized within each experiment. The conditioned media was aspirated from each flask, centrifuged at 4980×*g* for 10 mins and stored at −80°C. The samples were filtered through 0.2 µm Nanosep MF Centrifugal Devices with Bio-Inert® Membrane (Pall Scientific, USA). 50 µL of each filtered sample was analysed using the Bio-Plex Pro Human Cytokine 27-plex assay (Bio-Rad, USA), according to the manufacturer's instructions. The washing steps were performed using the Bio-Plex Pro II magnetic wash station and the data was acquired using the Bio-Plex 200 system with version 5.0 software (Bio-Rad, USA). The average concentration of each cytokine in the conditioned medium samples was calculated from the three replicate flasks. The results are presented as the mean ± standard deviation (SD) and analyzed using a two-tailed t-test with a p-value<0.05 being statistically significant.

### Preparation of MSCs and labeled MSCs for iTRAQ analysis

Four T175cm^2^ flasks of passage 3 cells from each treatment group (MSCs, MSCs labeled with M-SPIO particles and MSCs labeled with nanodiamonds as described earlier) were prepared. The adherent MSCs were washed with DMEM and liberated from the flask with TrypLE Express. The cells were collected in PBS, centrifuged at 1500×*g* for 5 mins, and the pellets were snap frozen in liquid nitrogen and stored at −80°C. Complete cell lysis and protein denaturation was achieved by adding 1% SDS and 100 mM TEAB to the thawed pellets. The samples were boiled, sonicated and centrifuged at 21 000×*g* for 5 mins. Pelleted cell debris and DNA were discarded and the supernatants containing the proteins were acetone precipitated then centrifuged at 4980×*g* for 6 mins. The resultant pellets were solubilized in 1% SDS and 100 mM TEAB followed by sonication, boiling and centrifugation at 21 000×*g* for 5 mins. Samples were processed through columns (micro Bio-spin, P6 in SSC; Bio-RAD, USA) according to the manufacturer's instructions. 100 µg of each of the 4 samples were reduced with tris(2-carboxyethyl)phosphine, alkylated with methyl methanethiosulfonate and digested with trypsin. The resultant peptides from each sample were labeled with 1 of the 4 iTRAQ labels (114, 115, 116 or 117) according to the manufacturer's instructions (AB Sciex, USA). This experiment was performed in triplicate.

The labeled samples were analysed using mass spectrometry as described previously [16]. Briefly, the labeled samples were combined, cleaned and fractionated by strong cation exchange chromatography, followed by nanoLC-ESI-MS/MS using a Qstar Elite mass spectrometer (AB Sciex, USA). The experimental nanoLC-ESI-ms/ms data were submitted to ProteinPilot V4.0 (AB Sciex, USA) for data processing using Homo sapiens species. Bias correction was selected. The detected protein threshold (unused ProtScore) was set as larger than 1.3 (>95% confidence). In each experiment, significant proteins were considered to have a fold change threshold of >1.2 and <0.8 and a p-value<0.05 in one or more experiments. Duplicate controls were run in each experiment to provide a list of experimental false positives that were then removed from the significant protein lists.

## Results

### Labeling of cells does not affect the defining characteristics of mesenchymal stem cells

As research into the properties and therapeutic potential of MSCs has grown, the number of studies published using different methods of MSC isolation, expansion and characterization have also increased. In an attempt to allow studies to be compared, the International Society for Cellular Therapy released a position statement outlining the three criteria required for a cell to be designated a MSC [18]. These criteria are that an MSC cell must display plastic adherence; have the ability to differentiate into cells of the mesenchyme lineage (bone, cartilage and adipose tissue); and lastly, express CD73, CD90 and CD105 on their cell surface whilst lacking expression of the hematopoietic markers CD34 and CD45 [18]. In this study we assessed morphology (including plastic adherence), differentiation potential and CD marker expression profiles of MSCs upon labeling with M-SPIO particles or nanodiamonds to determine if the extrinsic label had any effect on these MSC characteristics.

**Figure 1:**
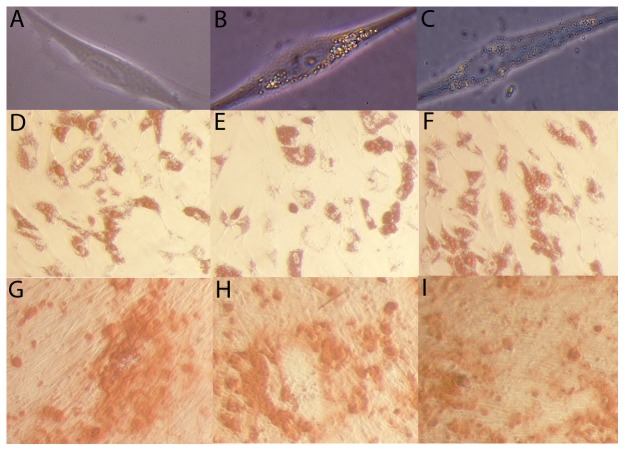
Morphology and differentiation potential of control and labeled adipose-derived mesenchymal stem cells. MSCs were cultured alone or in the presence of superparamagnetic iron oxide particles (M-SPIO) or nanodiamonds for 3 days. Control MSCs (A), ∼0.9 µm M-SPIO labeled MSCs (B) and ∼0.25 µm nanodiamonds labeled MSCs (C) all exhibited similar morphologies after adhering to plastic. No significant differences were observed between the adipogenic differentiation potential of control MSCs (D), M-SPIO labeled MSCs (E) and nanodiamond labeled MSCs (F) stained with Oil Red O. Control MSCs (G), M-SPIO labeled MSCs (H) and nanodiamond labeled MSCs (I) all displayed similar levels of osteogenic differentiation following staining with Alizarin Red to visualize calcium deposition.

**Figure 2:**
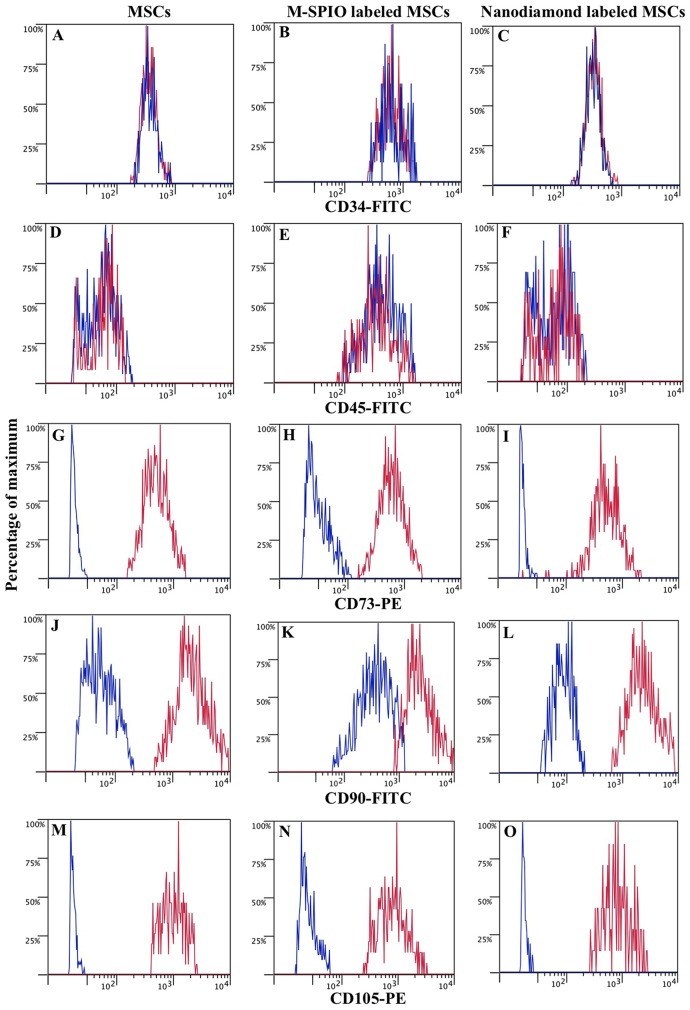
CD marker characterization of control and labeled adipose-derived mesenchymal stem cells. MSCs were cultured alone or in the presence of superparamagnetic iron oxide particles (M-SPIO) or nanodiamonds for 3 days. Control MSCs (A, D, G, J, M), MSCs labeled with M-SPIO particles (B, E, H, K, N) and MSCs labeled with nanodiamonds (C, F, I, L, O) were subsequently analyzed for their CD marker expression profiles. A comparison of unstained (blue) and stained cells (red) illustrated that control and labeled MSCs were negative for the hematopoietic markers CD34-FITC (A–C) and CD45-FITC (D–F) and positive for the stromal markers CD73-PE (G–I), CD90-FITC (J–L) and CD105-PE (M–O).

Figure 1A contains an image of a control MSC with the characteristic adherent fibroblastic shape. The labeling of MSCs with M-SPIO particles (Figure 1B) or nanodiamonds (Figure 1C) did not affect the morphology or the plastic adherence properties of the cells. Following treatment with a standard adipogenic differentiation media formulation, labeled MSCs stained positive for lipid accumulation at a level similar to control MSCs following Oil Red O staining (Figures 1D–F). Similarly, there was no difference in the level of calcium deposition following osteogenic differentiation and staining with Alizarin red between the labeled or control MSCs (Figures 1G–I). Lastly, the labeling of MSCs with M-SPIO particles or nanodiamonds did not affect the CD marker profiles of the MSCs as all cells expressed CD73, CD90 and CD105 on their surface and lacked the expression of CD34 and CD45 (Figure 2). These results confirm that the characteristics that are used to define MSCs were not affected by the labeling of MSCs with either the M-SPIO or nanodiamond particles.

### Labeling of MSCs does not alter their secretion profiles in vitro

To determine if the labeling of MSCs with M-SPIO particles or nanodiamonds affected the secretion capabilities of these cells, a panel of 27 cytokines and growth factors were measured in the conditioned medium from control and labeled cells. The secretion profiles obtained from control and labeled cells in each experiment are presented in Table 1. A two-tailed t-test was performed to determine if the concentration of each cytokine secreted by the control MSCs was significantly different from the levels produced by the labeled cells within that experiment. No cytokine was produced in significantly different levels by MSCs labeled with either M-SPIO particles or nanodiamonds when compared to the levels of the corresponding control MSCs.

**Table 1:** Cytokines secreted by control MSCs and MSCs labeled with M-SPIO particles or nanodiamonds.

Functional Category	Cytokine Name	Experiment 1		Experiment 2	
		Control MSCs	M-SPIO labeled MSCs	Control MSCs	Nanodiamond labeled MSCs
Pro-inflammatory	IFN-γ	66±7	65±9	74±11	76±1
	IL-1β	N.D	N.D	N.D	N.D
	IL-8	92±10	88±9	27±1	28±4
	IL-9	2±1	2±1	6±1	6±1
	IL-12	74±2	68±11	162±19	154±8
	IL-15	N.D	N.D	N.D	N.D
	IL-17	N.D	N.D	N.D	N.D
	TNF-α	4±1	3±1	2±1	3±0
Anti-inflammatory	IL-1ra	18±4	16±2	N.D	N.D
	IL-4	N.D	N.D	N.D	N.D
	IL-10	30±2	24±4	5±0	5±0
	IL-13	12±2	12±3	7±1	6±0
Chemokines	Eotaxin	N.D	N.D	2±1	2±4
	IP-10	N.D	N.D	13±11	14±3
	MCP-1	207±9	237±34	115±5	107±10
	MIP-1α	N.D	N.D	N.D	N.D
	MIP-1β	N.D	N.D	N.D	N.D
	RANTES	2±1	2±0	13±1	13±1
Growth Factors	bFGF	N.D	N.D	N.D	N.D
	G-CSF	3±1	3±1	17±4	15±1
	GM-CSF	N.D	N.D	17±4	20±2
	IL-7	2±0	2±0	8±0	7±1
	PDGF-bb	N.D	N.D	N.D	N.D
	VEGF	1998±121	1907±380	5359±166	4701±338
Dual Roles	IL-2	N.D	N.D	N.D	N.D
	IL-6	1893±81	1835±325	3503±18	3349±363

Adherent adipose-derived mesenchymal stem cells (MSCs) were obtained from a human lipoaspirate sample. The MSCs were cultured to passage 2 in Standard Media consisting of Dulbecco's modified eagle medium, 10% foetal bovine serum and 1% penicillin/streptomycin solution. MSCs were cultured alone or labeled with ∼0.9 µm superparamagnetic iron oxide particles (M-SPIO) or ∼0.25 µm nanodiamonds for 3 days. Conditioned medium was collected and analysed for a panel of 27 human cytokines and growth factors using Bio-Plex technology. The reported values are the average ± standard deviation for each cytokine in pg/mL (n = 3). A two-tailed t-test revealed no significant differences in the levels of any of the cytokines secreted by control and labeled cells in each experiment. N.D. refers to not detected.

### iTRAQ analysis of the intra-cellular proteome of control MSCs and MSCs labeled with M-SPIO particles or nanodiamonds

To supplement the differentiation potential, CD marker characterization and secretion profiling data, we chose to investigate if the intra-cellular proteome of MSCs was affected by the incorporation of M-SPIO or nanodiamond labels. Using the quantitative proteomic technique, iTRAQ, we identified a total of 3059 unique proteins from 32174 distinct peptides, which were identified from 85414 spectra using this method. Table 2 contains the protein identifications, percentage of protein coverage, number of unique peptides, and fold change of the protein in each comparison of the MSCs labeled with nanodiamonds versus control MSCs for each of the 5 proteins that showed a significant change (p value<0.05). Of these 5 proteins, 3 were up-regulated and 2 were down-regulated following the incorporation of nanodiamonds into MSCs when compared to control MSCs. However, for each of these 5 proteins, only 1 of the 3 replicate experiments were found to show significantly different levels (Table 2). This change in the levels of 5 intracellular proteins of MSCs labeled with nanodiamonds represents a change in only 0.16% of the proteins identified.

**Table 2:** Proteins differentially expressed in the intra-cellular proteomes of controls MSCs and MSCs labeled with nanodiamonds.

UniProt accession No.	Protein Name	MSCs labeled with nanodiamonds : MSCs		
		Experiment 1	Experiment 2	Experiment 3
Significant Up-Regulated Proteins				
P02765	Alpha-2-HS-glycoprotein	1.43	(1.39)	(0.98)
Q76M96	Coiled-coil domain-containing protein 80	(0.80)	1.31	(1.18)
Q00325	Phosphate carrier protein, mitochondrial	(0.88)	1.22	(0.90)
Significant Down-Regulated Proteins				
A0FGR8	Extended synaptotagmin-2	0.72	(1.02)	(1.00)
P46976	Glycogenin-1	(0.77)	(0.97)	0.67

Control adipose-derived mesenchymal stem cells (MSCs) or MSCs labeled with nanodiamonds were held in culture for 72 hours in Standard Media. The MSCs were subsequently collected, processed and the tryptic peptides were labeled with an isobaric tag for iTRAQ analysis using a Qstar Elite. The experiment was repeated in triplicate. A total of 3059 proteins were identified. The fold-change values of the proteins which changed by >1.2 and <0.8 and had a p-value<0.05 in one or more experiments have been included. The fold-change values that did not meet these criteria in each experiment are depicted in brackets. For each of these proteins, the UniProt identification number, protein name, percentage of protein coverage (and number of unique peptides contributing to the sequence coverage), and fold change between the proteins in the MSCs labeled with nanodiamonds versus control MSCs, for all replicates, are included.

A total of 17 proteins were found to be significantly differentially expressed in the intra-cellular proteome of MSCs labeled with M-SPIO particles when compared to control MSCs. Of these 17, 11 proteins were up-regulated and 6 were down-regulated following labeling of MSCs with M-SPIO particles (Table 3), representing a change in only 0.56% of the proteins identified. Interestingly, activated RNA polymerase II transcriptional coactivator p15 was the only protein found to be significantly differentially expressed in all three replicates.

**Table 3:** Proteins differentially expressed in the intra-cellular proteomes of controls MSCs and MSCs labeled with M-SPIO particles.

UniProt accession No.	Protein Name	MSCs labeled with M-SPIO particles:MSCs		
		Experiment 1	Experiment 2	Experiment 3
Significant Up-Regulated Proteins				
P30530	Tyrosine-protein kinase receptor UFO		1.45	(1.40)
P04114	Apolipoprotein B-100		(1.34)	1.45
P35625	Metalloproteinase inhibitor 3		1.38	(1.26)
P05067	Amyloid beta A4 protein		1.28	(1.15)
Q76M96	Coiled-coil domain-containing protein 80	(0.84)	1.23	1.26
Q13501	Sequestosome-1	(1.06)	1.26	(1.13)
Q14566	DNA replication licensing factor MCM6	(1.15)	(1.06)	1.25
P02765	Alpha-2-HS-glycoprotein	1.24	(1.15)	(0.96)
P05121	Plasminogen activator inhibitor 1	(1.01)	(1.09)	1.23
P33993	DNA replication licensing factor MCM7		(1.07)	1.22
P33991	DNA replication licensing factor MCM4		(1.05)	1.22
Significant Down-Regulated Proteins				
P17813	Endoglin	0.79	(0.93)	(0.86)
Q14498	RNA-binding protein 39	(0.85)	(0.83)	0.77
P16403	Histone H1.2	0.75	(0.71)	
P84103	Splicing factor, arginine/serine-rich 3	(0.47)	(0.71)	0.73
P16401	Histone H1.5	(0.76)	0.66	0.61
P53999	Activated RNA polymerase II transcriptional coactivator p15	0.52	0.65	0.60

Control MSCs or MSCs labeled with M-SPIO particles were held in culture for 72 hours in Standard Media. The MSCs were subsequently collected, processed and the tryptic peptides were labeled with an isobaric tag for iTRAQ analysis using a Q-Star Elite. The experiment was repeated in triplicate. A total of 3059 proteins were identified. The fold-change values of the proteins which changed by >1.2 and <0.8 and had a p-value<0.05 in one or more experiments have been included. The fold-change values that did not meet these criteria in each experiment are depicted in brackets. A blank space indicates that a relative fold-change was not obtained for the specified protein in that replicate. For each of these proteins, the UniProt identification number, protein name, percentage of protein coverage (and number of unique peptides contributing to the sequence coverage), and fold change between the proteins in the MSCs labeled with M-SPIO particles versus control MSCs, for all replicates, are included.

## Discussion

The labeling and tracking of MSCs to determine cell fate, level of engraftment and cell longevity following *in vivo* implantation is becoming critical for progress of the regenerative medicine field. There is considerable variability in the reports of MSC engraftment *in vivo*; the requirement for migration to the site of injury and the length of time that beneficial effects are observed after the implanted cells are not detectable [19]. The lack of reliable labels due to the dilution of the label below detectable levels over time and cellular transfer to host cells, coupled with the inability to track the cells repeatedly in real-time, are significant issues for cell labeling. Furthermore, before *in vivo* use, it is imperative to determine that the incorporation of the tracking particle/s into the MSCs, as well as other target cells, are not inducing any cellular changes that could affect their function and therefore the interpretation of the tracking results obtained. Consequently, we chose to investigate the effects of labeling with two different particle types, ∼0.9 µm superparamagnetic iron oxide particles with a dragon green fluorophore (M-SPIO) and ∼0.25 µm carboyxlated nanodiamonds, on the functionality of MSCs. The M-SPIO particles investigated are biocompatible and allow high resolution non-invasive real-time tracking of the labeled cells using MRI. Nanodiamonds are also attractive labels for long-term repetitive tracking experiments by fluorescence due to their unique photostability and the observation of little occurrence of exocytosis, even after 6 days of labeling [20].

Historically, stem cell research has focused on differentiating the stem cells into cells of the target tissue type, and their subsequent therapeutic use. Although differentiation is no longer considered the key mechanism behind the therapeutic effect of MSCs, the *in vitro* differentiation into cells of the mesenchymal lineage still remains as one of the three criteria required for a cell to be designated a MSC. Our results from this study, namely that MSCs labeled with either M-SPIO or nanodiamonds retain their ability to differentiate into cells of the mesenchymal lineage, as well as retain the MSC cell surface CD marker profile and plastic-adherence properties, indicates that the incorporation of the labels does not affect the properties of these cells which define them as MSCs. Similarly, Detante *et al.*, (2012) demonstrated that these stem cells characteristics were unaffected by M-SPIO labeling of human bone marrow derived MSCs [21] and Hinds *et al.*, (2003) demonstrated osteogenic differentiation was normal in porcine MSCs labeled with M-SPIO particles [22]. Previous functional studies, using 100–140 nm carboxylated nanodiamonds to label 3T3-L1 embryonic fibroblasts or 489-2 osteoprogenitors, reported that the nanodiamond particles did not interfere with cell growth or proliferation, or their adipogenic or osteogenic differentiation potential [20], [23], [24]. Although these studies used different types of stem cells and smaller nanodiamonds than we investigated, our results with ∼0.25 µm nanodiamonds are consistent with the general consensus in the literature that nanodiamond labels do not affect the phenotype or differentiation capacity of stem cells. However, the number of particles used for labeling is another important consideration. A study by Nohroudi *et al.*, (2010) demonstrated that labeling with 20 µg/mL of micron-sized SPIO did not affect MSC function or migration but labeling with higher concentrations (50 or 250 µg/mL) did alter both cell viability and migratory potential of the labeled cells [25]. In this study we labeled with 3.33 µg/mL of M-SPIO particles which had no effect on MSC cell characteristics.

Numerous studies have demonstrated that MSCs secrete large quantities of a variety of immuno-modulatory cytokines and growth factors of therapeutic importance. *In vivo* administration of MSCs has been shown to have beneficial effects on a variety of induced diseases in animals [26]–[29]. Furthermore, the administration of secretions produced from MSCs has been shown to have significant beneficial effects on wound healing [30], hindlimb [31] and cardiac ischemic injuries [32] and chemical burns to the cornea [33]. Consequently, the main mechanism of action that MSCs exert their therapeutic effect is through their ability to respond to the local environment and adjust their secretome to suppress T-Cell proliferation, decrease apoptosis and fibrosis and stimulate the endogenous cells to repair and regenerate the damaged tissue [9], [19], [34], [35]. In this study, we saw no significant changes in the secreted levels of 27 cytokines and growth factors by MSCs labeled with either M-SPIO particles or nanodiamonds when compared to control MSCs. These results demonstrate that the incorporation of these labels into the MSCs did not affect their ability to secrete cytokines and growth factors that are key to these cells achieving their therapeutic effect. Similarly, van Buul *et al.*, (2011) measured a small number of cytokines and growth factors secreted by bone marrow derived MSCs and illustrated that labeling these cells with superparamagnetic iron oxide particles (fermuoxides) did not affect their secretion profiles [36]. Furthermore, when immune cells recognize foreign material, they respond by secreting pro-inflammatory cytokines that result in recruitment of immune cells to the site [37]. As MSCs have the capability to phagocytose particles, the observation that the labeling of MSCs did not alter the levels of secreted cytokines suggests that these labels are not being flagged as foreign.

To supplement the secretion profile data, we chose to investigate whether the incorporation of the M-SPIO particles or nanodiamonds into MSCs had any negative effect on their intra-cellular proteomes that could affect their suitability as labels for *in vivo* tracking. Of the 3059 proteins identified, the 5 and 17 proteins differentially expressed in nanodiamond and M-SPIO labeled MSCs respectively, were not characteristic of stress or toxicity such as heat shock proteins [38]. This result indicates that the incorporation of these particles into MSCs did not have a negative effect on the cells. Furthermore, as some proteins were significantly differentially expressed in only one out of the three iTRAQ replicates, and with fold-changes of less than 2, the labels appeared to have minimal effects on the proteome and cellular functions of the MSCs.

The incorporation of M-SPIO particles into MSCs resulted in the differential expression of 17 intra-cellular proteins. Whilst the majority of these proteins were differentially expressed in only one of the three replicates, 3 were significantly differentially expressed in two or more of the replicates. Of these proteins, coiled-coil domain containing protein 80, which is involved in the regulation of adipogenic differentiation [39], was significantly up-regulated in two of the three replicates by approximately 1.25-fold, but down-regulated by 1.19-fold in the third replicate. No changes in other proteins related to adipogenesis were observed and the process of adipogenesis typically takes two to three weeks *in vitro*. The M-SPIO labeling was conducted in basal growth media and the experiment was concluded within three days. When M-SPIO labeled MSCs were maintained in basal media for three weeks there was no evidence of adipogenesis. Differentiation into any other cell type was only observed upon induction in specific media.

Histone H1.5 was significantly down regulated by approximately 1.55-fold in two of the three replicates of M-SPIO labeled cells. This protein forms part of the H1 complex of proteins that binds to the DNA as it enters and exits the nucleosome [40]. As this protein does not appear to be involved in the stress response mechanism of cells, the significance of the down regulation of this protein in the M-SPIO labeled cells is unclear. RNA polymerase II transcriptional coactivator p15, also referred to as PC4 was down-regulated by approximately two-fold in all three replicates. PC4 stimulates transcription [41] and also been reported to play a role in DNA repair in relation to oxidative stress [42]. The M-SPIO particles used in the study are polystyrene coated, however, they may have some iron oxide exposure on their surface. Oxidative stress does not seem to be a good explanation for the changes observed in PC4 because no other proteins in this pathway were detected with altered expression. In future experiments, to ensure the cells are not exposed to any iron, fully encapsulated M-SPIO particles could be used for labeling instead as they contain no iron oxide on their surface. Although we did observe some differentially expressed proteins in the intra-cellular proteomes of the M-SPIO labeled MSCs, the particles did not appear to be toxic to the cells or inducing any stress responses that could affect their suitability as a cell labeling agent.

Superparamagnetic iron oxide particles (SPIO) such as the M-SPIO particles used in this study are particularly attractive particles for cell labeling and *in vivo* tracking. This is primarily due to suitability for imaging using MRI. SPIO particles are considered ‘negative’ contrast agents as they reduce T_2_-weight spin-echo signal intensity and T_2_^*^-weighted gradient-echo magnetic resonance images [21], [43]. The effects on T_2_^*^-weighted MRI are thought to increase with increasing SPIO particle size [44]. This is important because two of the major drawbacks of using nanometer-sized SPIO particles for labeling are the requirement for millions of particles to concentrate in a specific point for detection and that mitosis results in a dilution of the label below detectable levels [43]. In contrast, the effects of micron-sized SPIO particles on T_2_^*^-weighted MRI enables the detection of a cell containing a single SPIO at ≈50-µm resolution [43]. Furthermore, there is also an increased risk with the use of nanometer-sized SPIO particles for labeling in that they may localize to intra-cellular organelles and significantly affect the functionality of the cells. As our long-term goal is to label and track cells following *in vivo* implantation into animals, we chose to investigate the effects of the micron-sized SPIO particles on the functionality of MSCs. Studies have successfully demonstrated MRI tracking of SPIO labeled MSCs following intra-cerebral grafting [45], [46] and intra-articular [36], intra-arterial [47] or intravenous injection [45], [47] in animal models. Furthermore, Shapiro *et al.*, (2004) demonstrated that despite injection of micron-sized SPIO particles into single-cell mouse embryos, the embryos developed normally [43]. This study and others have demonstrated no effect of labeling with SPIO on cell viability, phenotype or differentiation potential. The comprehensive proteomic analysis of the effect of M-SPIO particle labeling on the functionality of MSCs revealed a small number of up or down regulated proteins. However, the lack of stress response proteins or proteins indicating toxicity from the labeling in our study, coupled with the developmentally normal embryos following SPIO labeling reported by Shapiro *et al.*, (2004) suggests these labels are highly biocompatible and suitable for *in vivo* tracking. In addition to the ability to track cells in a longitudinal fashion using MRI, labeling with SPIO particles which are also fluorescent, such as the M-SPIO particles used in this study, allows the *ex vivo* detection and/or confirmation of particles by fluorescence microscopy.

Nanodiamonds are becoming increasingly attractive particles for cell labeling due to their luminescent photostability and biocompatibility. The fluorescent properties of these submicron-sized diamond particles stem from defects in their crystal lattice known as colour centres. Over 500 colour centres have been discovered and categorized [48], of which, the nitrogen-vacancy (NV) centre is the most widely studied. The NV centre consists of a substitutional nitrogen atom next to a vacant carbon site in the diamond lattice [49]. The NV centre is frequently incorporated naturally in the growth of the diamond but can also be artificially enhanced by nitrogen implantation [50], electron [51] or ion [52] irradiation methods. Incorporating additional NV centres within diamond particles thereby increases the brightness of the fluorescence. The abundant negatively charged NV centre can be excited between 480 and 580 nm with broad emission centered about 700 nm. This property is attractive for labeling applications because the emission can penetrate tissues and is beyond the range of cellular autofluorescence [53]. Furthermore, the quantum yield of the NV is close to unity [54] and, most importantly, it is photostable [55]. For this reason nanodiamonds are starting to be more appealing as fluorescent probes over conventional photobleaching dye molecules and fluorescent proteins (see review by Say *et al.*, (2011) [15].

### Conclusion

In this study we demonstrated that the incorporation of ∼0.9 µm M-SPIO particles or ∼0.25 µm carboxylated nanodiamonds into human MSCs had no detectable effect on the morphology, differentiation potential, CD marker expression or secretion capabilities of these cells. Although labeling of the MSCs did result in differential expression of a very small number of intra-cellular proteins when compared to control MSCs, these protein changes did not indicate that the particles were activating stress responses resulting in negative changes in the cellular proteome or function of these cells. From these functionally relevant tests we can conclude that the ∼0.9 µm M-SPIO particles and ∼0.25 µm carboxylated nanodiamonds used in this study are biocompatible with MSCs and can be used for *in vivo* cell tracking.
